# The Role of Sulfation in Nematode Development and Phenotypic Plasticity

**DOI:** 10.3389/fmolb.2022.838148

**Published:** 2022-02-10

**Authors:** Catia Igreja, Ralf J. Sommer

**Affiliations:** Max Planck Institute for Biology, Tübingen, Germany

**Keywords:** nematodes, sulfatases, *Caenorhabditis elegans*, *Pristionchus pacificus*, sulfotransferases, *eud-1*, developmental switch, developmental plasticity

## Abstract

Sulfation is poorly understood in most invertebrates and a potential role of sulfation in the regulation of developmental and physiological processes of these organisms remains unclear. Also, animal model system approaches did not identify many sulfation-associated mechanisms, whereas phosphorylation and ubiquitination are regularly found in unbiased genetic and pharmacological studies. However, recent work in the two nematodes *Caenorhabditis elegans* and *Pristionchus pacificus* found a role of sulfatases and sulfotransferases in the regulation of development and phenotypic plasticity. Here, we summarize the current knowledge about the role of sulfation in nematodes and highlight future research opportunities made possible by the advanced experimental toolkit available in these organisms.

## Introduction

Sulfation, sulfonation or sulfoconjugation, is an essential and ubiquitous biochemical reaction that modifies a wide range of xenobiotic and endogenous molecules. From bacteria to plants and animals, perturbation of the molecular processes that regulate the biologic ratio of sulfated and unconjugated substrates is likely to alter cellular and organismal physiology (Leung et al., 2016; Langford et al., 2017; Gunal et al., 2019). The sulfation pathways are catalyzed by two types of enzymes, the sulfotransferases (sulfuryltransferases) and the sulfatases. The sulfotransferases transfer a sulfuryl group (SO_3_) from the universal donor PAPS (3′-phosphoadenosine 5′-phosphosulfate) to the hydroxyl or amino group of numerous compounds (Strott, 2002; Coughtrie, 2016). The presence of the charged sulfate group changes the physiochemical properties (water solubility and conformation) of the acceptor molecules and has therefore a major role in the detoxification and elimination of various xenobiotics (Strott, 2002; Leung et al., 2016). Multiple endogenous compounds including carbohydrates, lipids, proteins and hormone precursors (steroids) are also modified by sulfotransferases. In their case, sulfation has significant influence on the biological activity of the modified molecules and consequently in multiple biological processes (for details see (Strott, 2002; Mueller et al., 2015; Leung et al., 2016)).

The sulfatases, on the other hand, hydrolyze the sulfate ester bonds to the unconjugated form of the substrate (Hanson et al., 2004). These hydrolytic enzymes are part of the alkaline phosphatase superfamily and share a post-translational modification that greatly enhances enzymatic activity: a cysteine or serine in the catalytic center is converted to a formylglycine (Schmidt et al., 1995; Dierks et al., 1997; Dierks et al., 1998a; Dierks et al., 1998b). This review elaborates on the players, molecular mechanisms and roles of the sulfation pathways in *Caenorhabditis elegans* and *Pristionchus pacificus*. We especially highlight the use of these nematode model organisms to investigate the poorly understood relevance of the sulfation pathways in organismal development. Finally, the emerging topics and questions on sulfation in nematodes are discussed.

### The Nematode Model Organisms: *C. elegans* and *P. pacificus*

**FIGURE 1:**
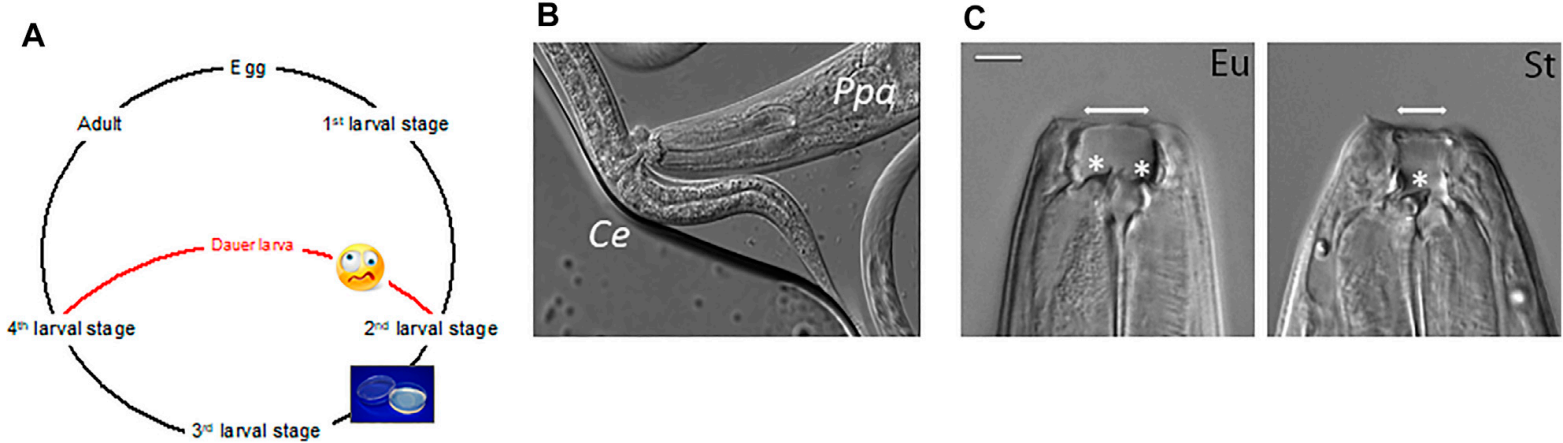
Nematode development and plastic traits. **(A)** Schematic life cycle of *Caenorhabditis elegans* and *Pristionchus pacificus,* including four postembryonic larval (juvenile) stages separated by molts. Worms can either directly develop into a reproductive adult if conditions are favorable and food is available (symbolized by plates with food), or indirectly through a stress-resistant and long-lived dauer larval stage if environmental conditions are unfavorable. **(B)** Predatory behavior of *P. pacificus* (*Ppa*): the eurystomatous morph of the worm is able to devour *C. elegans* (*Ce*) larvae. **(C)** Differential interference contrast (DIC) image depicting the mouth-form of eurystomatous (Eu) and stenostomatous (St) worms. The eurystomatous morph has two teeth (asterisks) and a wide buccal cavity whereas the stenostomatous morph shows only one tooth in a narrow buccal cavity (arrows). Bar = 10 μm.

Nematodes, or roundworms, are one of the largest animal phyla and are found in all ecosystems: marine, fresh-water and soil (van den Hoogen et al., 2019). In addition, many nematode species are parasites of plants, animals, life stock or humans, resulting in their economic importance for agriculture, breeding and health (Lee et al., 2002). Most of these parasites are hard to culture in the laboratory, whereas some free-living species are among the most rapidly reproducing and most easily culturable species of all metazoans. One example is *Caenorhabditis elegans,* which was introduced as a potential model organism by Sydney Brenner in the 1960ies (Brenner, 1974). Building on a unique set of life history traits and a growing functional toolkit, the ‘*C. elegans* research community’ has grown rapidly to make fundamental discoveries in all areas of the life sciences. *C. elegans* can be grown on small agar plates in the laboratory, feeds on *Escherichia coli* and completes its life cycle in 3 days at 20 °C (Figure 1A). This species reproduces primarily by selfing of hermaphrodites, which produce around 200–300 sperm during larval development before switching to a female fate with oocyte production as adults. Consequently, the progenies of hermaphrodites are genetically identical and the resulting isogenic propagation represents a unique advantage among all animal model organisms. Original unbiased forward genetic screens have identified many mutants essential for animal survival, egg-laying, neurogenesis or life span (Wood and Researchers, 1988). With *RNA interference* and CRISPR/Cas9-based genome engineering being implemented, large scale reverse genetic screens have been performed aiming at the analysis of the knock-down or knock-out of all genes in the genome (Fire et al., 1998; Dickinson and Goldstein, 2016). Indeed, when the *C. elegans* genome was fully sequenced, the 100 Mb genome was shown to encode around 20,000 genes with several thousands of them being functionally characterized (Consortium, 1998). Together, these features made *C. elegans* one of the most important study systems in modern biology.

The related nematode *Pristionchus pacificus* shares many of these features with *C. elegans* and similarly builds on a short generation time (4 days, 20°C), self-fertilizing hermaphroditism, forward and reverse genetic tools and a fully sequenced genome (Sommer and Sternberg, 1996; Dieterich et al., 2008; Han et al., 2020). *P. pacificus* and *C. elegans*, like many nematodes, can undergo direct and indirect development (Figure 1A). Under favorable environmental conditions and the availability of food, they undergo direct development, rapidly proceeding through four juvenile (larval) stages to become adults. In contrast, in the absence of food and other unfavorable circumstances, they arrest in a long-lived and stress-resistant “dauer” stage, which is adapted to survive harsh environmental conditions (Figure 1A) (for review see (Sommer and Mayer, 2015)). Besides these conserved features, *P. pacificus* differs from *C. elegans* with regard to several morphological and organismal features that turned out to be under the control of sulfation-mediated processes. Specifically, while *P. pacificus* can be cultured on *E. coli* as single food source indefinitely, it is a potential predator of other nematodes (Figure 1B). It forms teeth-like denticles in its mouth that are unknown in *C. elegans* or most other free-living nematodes (Figure 1C) (Bento et al., 2010). These teeth come in two alternative mouth-forms, representing an example of developmental (phenotypic) plasticity (Sommer, 2020). Worms can either form a “eurystomatous” (Eu) morph with two teeth and a wide buccal cavity, or, alternatively, a “stenostomatous” (St) morph with a single tooth and a narrow opening (Figure 1C) (Ragsdale et al., 2013). While both morphs can feed on bacteria, only the Eu form is a potential predator with associated self-recognition systems preventing the killing of kin (Lightfoot et al., 2019). The role of sulfation enzymes, the sulfatase *eud-1* and the sulfotransferase *seud-1/sult-1,* in the regulation of mouth-form plasticity were discovered through unbiased forward genetic screens and will be described below (Ragsdale et al., 2013; Bui et al., 2018; Namdeo et al., 2018).

### Sulfotransferases in Nematodes

Eukaryotes express cytosolic and membrane-bound sulfotransferases. Cytosolic sulfotransferases (termed SULTs) sulfate xenobiotics and small molecules like neurotransmitters and hormones. The membrane-bound sulfotransferases reside in the Golgi and mediate the post-translational sulfation of endogenous macromolecules (proteins, lipids and glycosaminoglycans) in the secretory pathway (Bojarova and Williams, 2008; Leung et al., 2016). Despite low sequence conservation, sulfotransferases possess a conserved protein structure and catalytic properties, which include the binding to the PAPS cofactor. However, sulfotransferases show broad substrate specificity and may sulfate an unlimited number of substrates (Mueller et al., 2015; Langford et al., 2017). The human genome contains 13 cytoplasmic and 39 membrane-bound sulfotransferases (Langford et al., 2017; Gunal et al., 2019). Based on published literature and Wormbase (version WS282), Table 1 lists the cytosolic and membrane-bound sulfotransferases currently identified in *C. elegans* (36), *P. pacificus* (18) and *Strongyloides ratti* (48), a common gastro-intestinal parasitic worm of the rat (Streit, 2008).

**TABLE 1:** Cytosolic and membrane-bound sulfotransferases in nematodes and corresponding human orthologues.

	*H. sapiens*	*C. elegans*	*P. pacificus*	*S. ratti*	
Cytosolic SULTs	SULT1B1 SULT2A1 SULT2B1	SSU-1	SULT-1 (PPA12547)	SRP02937	1-hydroxypyrene sulfotransferase activity and arylsulfotransferase activity; cytosolic
				SRP02634	
				SRP05555	
				SRP07442	
			SULT-2 (PPA36905)	SRP08825	
				SRP08910	
				SRP09379	
			SULT-3 (PPA06620)	SRP09540	

			SULT-4 (PPA22156)		
			SULT-5 (PPA41942)		
	SULTs		PPA20882		
			PPA16538		
			PPA00693		
			PPA22912		
Protein-tyrosine sulfotransferase	TPST1	TPST-1	TPST-1 (PPA25491)	SRP01681	Golgi; Tyrosylprotein sulfotransferase 1
				SRP00103	
	TPST2				
	TPST1	TPST-2	—	SRP09791	Protein-tyrosine sulfotransferase; Golgi
	TPST2				
Heparan sulfate proteoglycan biosynthetic process	NDST1	HST-1	HST-1 (PPA38610)	SRP03281	Heparan sulfate-glucosamine N-sulfotransferase and deacetylase activity
	NDST2				
	NDST4				
	HS2ST1	HST-2	HST-2 (PPA21724)	SRP00819	Heparan sulfate-glucosamine 2-O-sulfotransferase activity
	HS3ST5	HST-3.1 (transmembrane protein)	HST-3.1 (PPA39158)	SRP08308	Heparan sulfate-glucosamine 3-sulfotransferase 1 activity
	HS3ST6	HST-3.2 (secreted protein)	HST-3.2 (PPA32231)	SRP04407	Heparan sulfate-glucosamine 3-sulfotransferase 1 activity
	HS6ST1	HST-6	HST-6 (PPA28737)	SRP12180	Heparan sulfate- 6-O-sulfotransferase activity
	HS6ST3				
Chondroitin sulfate biosynthetic process	CHSTs	B0273.115	PPA19622	SRP00399	Chondroitin 4-sulfotransferase activity
				SRP00911	
		C31B8.9		SRP01497	
				SRP01663	
		C54F6.3		SRP02672	
				SRP02896	
				SRP03731	
				SRP03749	
		F01D5.10		SRP04122	
				SRP04305	
				SRP04354	
				SRP04890	
				SRP05289	
				SRP05718	
		F17B5.4		SRP06402	
				SRP06805	
				SRP06991	
		F25E5.2		SRP07055	
				SRP07472	
				SRP08449	
		F36D1.8		SRP08386	
				SRP08576	
				SRP09039	
				SRP09641	
		F40C5.1		SRP09844	
				SRP10988	
				SRP11197	
				SRP11358	
				SRP11499	
		F49D11.3			
		F49D11.6			
		F55B12.2		SRP11527	
		F56H6.4			
		F56H6.13			
		F59D12.3			
		K06H6.5			
		K07H8.8			
		T15D6.1			
		T24A6.16			
		T27C5.12			
		ZK1025.2			
		ZK1025.8			
		CHST-1	PPA16534	SRP10755	
		C18B2.1			
		C18B2.2			
		F20C5.7			
				SRP09150	
		T09E11.3			
		Y87G2A.16			
		Y48G1BL.7	PPA09342		

Protein searches were performed on the *C. elegans* genome at Wormbase (version WS282), the *P. pacificus* transcriptome (El_Paco_V3 annotation), and the *S. ratti* genome (PRJEB125 version WBPS16). Human orthologous proteins were assigned according to Wormbase curation and/or literature. Note that nematode nomenclature of sulfotransferases follows the standard nomenclature rules of *C. elegans,* for example *ssu*-1, follows the 3-letter code of genetic mutants and was isolated as a *S*uppressor of *S*tomatin mutant *U*ncoordination. All other genes, were described based on sequence homology and often related to the corresponding enzymes in humans.

### Nematode Cytosolic Sulfotransferases

*ssu-*1 (suppressor of stomatin mutant uncoordination) is the only member of the cytosolic sulfotransferase gene family present in the genome of *C. elegans* (Carroll et al., 2006; Hattori et al., 2006; Mizuguchi et al., 2009). Orthologous proteins include the human SULT1B1, SULT2A1 and SULT2B1 enzymes (Wormbase curation). This sulfotransferase is expressed throughout development, particularly in embryos and adult stages, but shows increased levels in dauer larvae. Recombinant protein is able to sulfate simple phenol substrates, such as bisphenol A (Hattori et al., 2006). Expression of SSU-1 has also been detected in the amphid sensory neurons located in the head of the worm where it is hypothesized to modify endocrine signals (Carroll et al., 2006). A recent study observed that mutant *ssu-1* animals failed to arrest development in response to osmotic stress. The authors suggest that in the sensory neurons SSU-1 sulfates a ligand of the nuclear hormone receptor NHR-1 to activate a transcriptional response to osmotic stress and induce developmental arrest (Burton et al., 2018).

Five paralogs of *C. elegans* SSU-1 are found in *P. pacificus*, termed SULT-1 to SULT-5 (Table 1) (Namdeo et al., 2018). CRISPR/Cas9-mediated knockout of these cytosolic sulfotransferases and a genetic screen for suppressors of an all-stenostomatous mutant of *P. pacificus* has identified a role for SULT-1 (PPA12547) in the regulation of mouth-form plasticity (see details below) (Bui et al., 2018; Namdeo et al., 2018). Additional SULTs can also be found in *P. pacificus* (4) and *S. ratti* (8). Their biological role remains unknown.

### Nematode Membrane-Bound Sulfotransferases

#### Tyrosylprotein Sulfotransferases

The genomes of nematodes also contain genes encoding TPSTs (Table 1) (Namdeo et al., 2018), integral membrane glycoproteins of the *trans*-Golgi network that post translationally sulfate tyrosine residues of soluble and membrane proteins of the secretory pathway. Sulfation is the most common modification of tyrosine residues and regulates biological activity and correct cellular processing (Monigatti et al., 2006; Stone et al., 2009; Yang et al., 2015). Two types of tyrosylprotein sulfotransferases (TPST1 and TPST2) with distinct substrate preference are present in animals; *P. pacificus* lacks the second TPST gene and *S. ratti* encodes three TPST enzymes (Table 1) (Beisswanger et al., 1998; Ouyang and Moore, 1998; Mizuguchi et al., 2009; Namdeo et al., 2018). In *C. elegans*, TPST-1 activity is required for cuticle organization as it controls collagen secretion (Kim et al., 2005; Kim et al., 2010).

#### Carbohydrate Sulfotransferases

Sulfotransferases also extensively modify carbohydrates/sugars along the secretory pathway. Carbohydrate sulfoforms function on the cell surface and in the extracellular matrix where they provide structural support and mediate communication between cells and the surrounding environment (Bowman and Bertozzi, 1999). Sulfation of carbohydrates creates structural variety, enables electrostatic interactions and generates ligands for specific receptors. The biological significance of the sulfation of carbohydrates is therefore broad as it regulates multiple processes, such as organ development, extracellular signaling, and inflammation, among others (Bowman and Bertozzi, 1999; Strott, 2002). Carbohydrate sulfotransferases are integral membrane enzymes of the Golgi apparatus that sulfate glycans attached to lipids and proteins (Bowman and Bertozzi, 1999; Strott, 2002). Recognizable carbohydrate sulfotransferases in nematodes modify the scaffold of proteoglycans (glycosaminoglycan chains constituted by repeating disaccharide units attached to core proteins) to produce variable sulfoforms. Two groups of carbohydrate sulfotransferases are present in *C. elegans*, *P. pacificus* and *S. ratti*: heparan sulfate (HS) and chondroitin sulfate (CS) sulfotransferases (Table 1). Sequence homology searches revealed 28 CS-6-O-sulfotransferases-like genes (CHST) in *C. elegans* (Mizuguchi et al., 2009), with three orthologs in *P. pacificus* (Namdeo et al., 2018) and 32 in *S. ratti* (Table 1). Five HS-sulfotransferases (HSTs) are also encoded in the genomes of the three nematodes (Mizuguchi et al., 2009; Namdeo et al., 2018) (Table 1).

a) Biological roles of CS-sulfotransferases in *C. elegans*

Chondroitin sulfate chains are composed of disaccharide units of glucuronic acid (GlcUA) and iduronic acid-N-acetylgalactosamine (GalNAc) heterogeneously sulfated (Sugahara et al., 2003). Sulfation of CS occurs on the 4- and 6-hydroxyl groups of GalNAc and on the 2-hydroxyl group of GlcUA. CS is the main structural component of the extracellular matrix in the brain and cartilage. In addition, CS sulfoforms regulate cell signaling through specific receptor-ligand binding influencing processes such as neuronal and skeletal development, and infection by pathogens (Bowman and Bertozzi, 1999; Sugahara et al., 2003).

Nematodes produce vast amounts of non-sulfated chondroitin (Yamada et al., 1999; Toyoda et al., 2000; Olson et al., 2006; Lawrence et al., 2008), which in *C. elegans* is crucial for cell division during embryogenesis and vulva morphogenesis (Herman et al., 1999; Hwang et al., 2003; Mizuguchi et al., 2003). Mutations or RNAi depletion of the nematode chondroitin synthase gene are proposed to decrease chondroitin hydration activity controlling tissue osmotic pressure (structural role) and/or disrupt cell signaling (Hwang et al., 2003; Mizuguchi et al., 2003). Although the presence of CS in *C. elegans* has been controversial, several studies detected different sulfoforms in the worms (Schimpf et al., 1999; Beeber and Kieras, 2002; Dierker et al., 2016). Recently, the first CS sulfotransferase enzyme has also been characterized in *C. elegans*, CHST-1 (Table 1) (Dierker et al., 2016), suggesting that sulfate CS has a biological role also in nematodes. Recombinant CHST-1 utilizes PAPS to sulfate a chondroitin substrate, and the levels of 4-*O*-sulfation in chondroitin are dramatically reduced in the *chst-1* mutant strain (Dierker et al., 2016). Whether the 26 other putative CS-sulfotransferases present in *C. elegans* genome encode for functional sulfotransferases with effects on CS sulfation and worm biology remains unclear, similar to the function of CS sulfotransferases in other nematodes.

b) Biological roles of HS-sulfotransferases in *C. elegans*

HS molecules are linear glycosaminoglycan polysaccharides composed of glucuronic acid (GlcUA) and iduronic acid-*N*-acetylglucosamine (GlcNAc) repeat units covalently bound to a protein core. As components of extracellular matrices and cell surfaces, HS function in a wide range of cellular processes, are essential in development and homeostasis, and have several implications in disease (Bowman and Bertozzi, 1999; Strott, 2002; Lindahl and Li, 2009). Three different types of HS are present in cells: syndecans (transmembrane proteins), glycosylphosphatidylinositol (GPI)-anchored proteins (e.g., glypicans) and secreted molecules (e.g., perlecan, agrin, and collagen XVIII) (Lindahl and Li, 2009). The genome of *C. elegans* contains single orthologues of these major proteoglycans (syndecan/F57C7.3, glypican/F59D12.4, perlecan (*unc-52*) and agrin/F41G3.8) (Rogalski et al., 1993; Hutter et al., 2000; Ackley et al., 2001; Bulow and Hobert, 2004; Minniti et al., 2004; Gumienny et al., 2007). After synthesis in the Golgi complex, heparan sulfate polymers are highly modified by deacetylation, sulfation and epimerization catalyzed by specific HS modifying enzymes at selective positions. The bifunctional enzyme *N*-deacetylase/*N*-sulfotransferases (Ndst) removes the acetyl group from GlcNAc and substitutes it with a sulfuryl group (Wei et al., 1993). The reactions catalyzed by Ndst allow the subsequent epimerization of glucuronic acid to iduronic acid and sulfation of HS, as the other enzymes recognize the *N*-sulfate groups on the molecule (Habuchi, 2000). The HS *C*-5 glucuronyl-epimerase is responsible for epimerization, while the HS 2-*O*, HS 6-*O*, HS 3-*O* sulfotransferases add SO_3_ at the C2 hydroxyl group of hexuronic acids and the C6 or C3 of glucosamine units, respectively (Habuchi, 2000; Turnbull et al., 2001; Lindahl and Li, 2009). Orthologues of all of these enzymes are present in nematodes (Table 1). These modifications increase the structural diversity of HS and influence cellular processes such as cell migration, axon guidance and pathogen infection as they are binding sites for multiple ligands, receptors, growth factors, enzymes, extracellular matrix proteins and adhesion proteins found in pathogens. These interactions are specific and perform regulatory roles (Turnbull et al., 2001; Lindahl and Li, 2009).

Work in *C. elegans* has increased the understanding of the relationships among HS modifications and specific proteoglycans in defined biological processes. The HS-modifying enzymes have differential effects in the development of the nervous system. For example, the modifications catalyzed by *C*-5 glucuronyl-epimerase (*hse-5*), HS 2-*O* (*hst-2*) and HS 6-*O* (*hst-6*)-sulfotransferases have been described to differentially regulate specific signaling pathways controlling axonal development (Bulow and Hobert, 2004). Efficient Robo, Ephrin and possibly Integrin-associated signaling pathways are dependent on distinct HS modifications to properly guide axon patterning in specific cellular contexts. These signaling pathways act in concert with precise modifications on components of the extracellular matrix to organize the complexity of the nervous system (Bulow and Hobert, 2004). In addition, the activity of HST-6 has been shown to modulate the function of anosmin (Kallman protein, *kal-1*), a secreted neural cell adhesion molecule with roles in axonal growth (Bulow et al., 2002). Parallel HS modifications also participate in the migration of *C. elegans* hermaphrodite specific neurons (HSNs), the pair of neurons that coordinates contraction of the vulval muscles to initiate egg laying (Desai et al., 1988; Kinnunen et al., 2005; Kinnunen, 2014). The migration of these neurons that are born in the posterior towards the mid body region is supported by HST-6-dependent sulfation of syndecan and HST-2-mediated sulfation of glypican. Genetic elimination of *hst-2* (expressed in the muscle and hypodermis) and *hst-6* (predominantly expressed in neurons) causes severe defects in the migration of the HSNs (Kinnunen, 2014). RNAi of the *hst-2* gene in *C. elegans* also causes aberrant morphology of the gonad and defects in egg laying, suggesting that sulfated HS chains are required for normal development and function of the reproductive tissues (Turnbull et al., 2003; Kinnunen et al., 2005).

The function of HST-3 enzymes has likewise been investigated in *C. elegans*. In *loss-of-function* mutants of *hst-3.1* and *hst-3.2,* worm viability and overall neuronal development are not affected. Yet, HS-3-*O*-sulfation has a specialized role in HSNs. Together with KAL-1, both enzymes are important for the branching of neurites in these neurons (Tecle et al., 2013). Forward genetic screens, have also identified *hst-6* and *hst-3.2* as modifiers of *kal*-1-dependent neurite branching of AIY neurons, a left/right pair of interneurons with axons that innervate the nerve ring in the head of the worm (Diaz-Balzac et al., 2014). Thus, different HS modifications regulate *kal-1*-dependent branching in distinct tissues. As KAL-1/anosmin is the underlying gene causing hereditary Kalmman syndrome in humans (Franco et al., 1991; Legouis et al., 1991), these studies highlight the importance of using nematode model systems to understand the role of HS in development and disease-associated processes.

Mating behavior between hermaphrodites and males is also controlled by sulfation of HS molecules. During mating, the male will press its tail against the body of the hermaphrodite and move backwards in search for the vulva (Liu and Sternberg, 1995). Abnormal response to mate contact has been observed in *hst-2*, *hst-3.1* and *hse-5* mutant worms, suggesting that HS modifications are required for the response to hermaphrodite contact by the male worm (Lazaro-Pena et al., 2018). Specifically, 3-*O*-sulfation of glypicans regulates synaptogenesis in neurons controlling male mating behavior, the B-type ray sensory neurons (Liu and Sternberg, 1995; Koo et al., 2011; Lazaro-Pena et al., 2018).

Finally, HS modifications have also been shown to regulate cilia structure in *C. elegans* (Acker et al., 2021). The cilia protrusions are organized in highly compartmentalized microtubule-based domains and disruption of their composition and structure results in ciliopathies (Reiter and Leroux, 2017). Null alleles of *hst-3.1* show defects in the localization of complement factor H (CFH) to the cilia of mechanosensory neurons. A potential interaction of CFH with modified HS appears to control cilia structural organization. HST-3.1 is proposed to modify the HS chain of proteoglycans that recruit CFH in the mechanosensory neurons. In the absence of this modification, CFH is not properly localized in the cilia and the animals show defects in mechanosensory neuron function (Acker et al., 2021). The role of HS modifications in cilia structure is another example of how studies in nematodes increase our knowledge on disease associated processes, since CFH is a major risk factor for age-related macular degeneration and blindness in humans (Edwards et al., 2005; Hageman et al., 2005; Haines et al., 2005; Klein et al., 2005; Zareparsi et al., 2005).

In summary, the differential modification of molecules of HS provide a “code” that mediates the selective interaction with a set of signaling factors and molecules that define the migration, patterning and function of different neurons (Bulow and Hobert, 2004; Lindahl and Li, 2009; Kinnunen, 2014).

### Sulfatases in Nematodes

From prokaryotes to eukaryotes sulfatases are highly conserved in sequence, structure and mechanism of action (Hanson et al., 2004; Ghosh, 2007). They share a highly conserved N-terminal region containing two consensus sulfatase motifs. The first motif is characterized by the conserved sequence C/SxPxRxxxxTG (x is any amino acid) and is crucial to generate the post-translationally modified active-site aldehyde residue, the α-formylglycine (FGly; 2-amino-3-oxypropanic acid) (Knaust et al., 1998; Dierks et al., 1999). The second sulfatase signature motif is a 12-mer sequence GY/VxS/T-xxxGKxxH. The Lys and His residues are part of the active-site and required for catalysis (Waldow et al., 1999). The C-terminal region of sulfatases is the most diverse; however, it is proposed that this region provides substrate specificity (Hanson et al., 2004). Although a large number of eukaryotic sulfatases are active on small aromatic sulfates (aryl substrates) *in vitro* and thus classified as arylsulfatases, many sulfatases show strong substrate specificity, specific subcellular location and optimal activity under defined conditions (Hanson et al., 2004). Sulfatase genes encode proteins with a wide range of substrate specificity but little functional redundancy, and deficiency in a single sulfatase can lead to unique disorders and developmental defects in animals (Langford et al., 2017).

Eukaryotic sulfatases are targeted to the secretory pathway. In the endoplasmic reticulum (ER), the sulfatases are modified by glycosylation and FGly is generated (Dierks et al., 1997; Dierks et al., 1998a; Hanson et al., 2004). They can then remain in the ER, be secreted, or transported to other subcellular compartments, such as the Golgi complex and the lysosome (Table 2) (Hanson et al., 2004). The steroid sulfatase (STS, human arylsulfatase C) is integrated into the membrane of the ER or Golgi apparatus (Willemsen et al., 1988; Hernandez-Guzman et al., 2003) where it plays an important role in the regulation of endocrine responses and xenobiotic metabolism. STS is the main enzyme involved in steroid desulfation and regulates hormone levels by removing sulfate from precursors, such as estrone sulfate and dehydroepiandrosterone (DHEAS), and originating active steroid hormones (for review see (Mueller et al., 2015)). Lysosome-located sulfatases (human arylsulfatases A and B) participate in the catabolism of defective and unwanted sulfated glycolipids (sulfatides) and proteoglycans that are imported into this organelle by endocytosis. Lysosome storage disorders are therefore a consequence of lysosome-sulfatase deficiency (Langford et al., 2017). The secreted sulfatases, known as Sulfs, remove the 6-*O*-sulfate groups of the glucosamine units of HS regulating multiple developmental processes (Hanson et al., 2004; Bojarova and Williams, 2008; Langford et al., 2017).

**TABLE 2:** Sulfatases in nematodes and corresponding human orthologues.

	*H. sapiens*	*C. elegans*	*P. pacificus*	*S. ratti*	
Heparan sulfate proteoglycan biosynthetic process	SULF1	SUL-1	SUL-1 (PPA46687)	SRP07877	Extracellular sulfatases; Removal of 6-*O*-sulfate from heparan sulfate
	SULF2				
	ARSL/E	SUL-2	SUL-2.1 (PPA21290) SUL-2.2.1 (PPA06135)EUD-1 (PPA43535)	SRP01584	Golgi; ER (steroid sulfates)
	STS				
	GALNS				
					Lysosome (chondroitin
					sulfate, keratan sulfate)
	ARSB	SUL-3	SUL-3 (PPA23475)	SRP06160	Lysosome
	ARSI				
	ARSJ				
					ER, secreted

Protein searches were performed on the *C. elegans* genome at Wormbase (version WS282), the *P. pacificus* transcriptome (El_Paco_V3 annotation), and the *S. ratti* genome (PRJEB125 version WBPS16). Human orthologous proteins were assigned according to Wormbase curation and/or literature.

Nematodes also contain multiple sulfatase genes: three in *C. elegans* and *S. ratti*, and five in *P. pacificus* (Table 2). Sequence-wise Wormbase indicates that SUL-1 proteins are orthologues of the secreted SULF-1 and SULF-2 sulfatases that act on HS. In contrast, SUL-2 proteins show similarity with the human arylsulfatases A, B that participate in the degradation of cerebroside-3-sulfate and keratan/dermatan sulfate or CS, respectively, the steroid sulfatase (STS) and N-acetylglucosamine-6-sulfatase (GALNS) (Ragsdale et al., 2013; Perez-Jimenez et al., 2021), another lysosome-resident protein involved in the catabolism of HS (Schlotawa et al., 2020). SUL-3 proteins are most similar to the lysosome sulfatase human arylsulfatase B that participates in the degradation of keratan/dermatan sulfate and CS, or the potentially secreted arylsulfatases I and J with currently uncharacterized substrates. The biological function, localization and substrates of the nematode sulfatases are largely unexplored. To date, efforts to understand desulfation in worms have been restricted to *C. elegans* SUL-2 and *P. pacificus* EUD-1 (SUL-2 paralog). The role of EUD-1 in the regulation of mouth-form and predation is discussed below.

*C. elegans* SUL-2, the worm steroid sulfatase (Perez-Jimenez et al., 2021), is expressed in sensory neurons of the head of the worm where it is proposed to regulate the levels of steroid hormones. Worms with *loss-of-function* mutation of *sul-2* or treated with the steroid sulfatase (STS) inhibitor STX64 have higher levels of sulfate steroid hormones and increased longevity. The authors of the study hypothesize that sulfated steroid hormones produced in the gonads of the worm may act in response to environmental cues, such as nutrient availability, to alter neurotransmission and promote longevity. Furthermore, higher levels of sulfated hormones as a result of reduced SUL-2 activity, or treatment of worms with sulfated steroid hormones, reduced protein aggregation and proteotoxic stress in models of Parkinson and Huntington neurodegenerative disorders. These data indicate that inhibitors of STS activity and sulfated steroid hormones have great potential in the treatment of aging-related diseases (Perez-Jimenez et al., 2021). In the same study, genetic ablation of *sul*-1 and *sul*-3 had no effects on lifespan.

The post-translational modification that enhances sulfatase activity is catalyzed by an endoplasmic reticulum (ER)-resident protein, the FGE-generating enzyme (FGE, sulfatase modifying factor 1, *SUMF1*) (Dierks et al., 1998a; Cosma et al., 2003; Dierks et al., 2003; Appel and Bertozzi, 2015). The FGly modification is essential for sulfatase activity (Dierks et al., 1998b; Recksiek et al., 1998) and is able to greatly stimulate catalytic proficiency, which can reach a rate enhancement (k_cat_/k_uncat_) of 10^26^ for alkylsulfates (most efficient enzymes known to date) (Edwards et al., 2012). In humans, lack of FGly modification in sulfatases due to mutations in FGE that alter its activity causes multiple sulfatase deficiency (Schmidt et al., 1995; Schlotawa et al., 2020). FGE orthologues are found in prokaryotes and eukaryotes. Yet, homology searches indicate that FGE homologs are not present in plants (which also lack sulfatases), nematodes and fungi, although these last two groups of organisms contain cysteine-type sulfatase genes (Landgrebe et al., 2003; Appel and Bertozzi, 2015). This observation is suggestive of a different FGly-generating system in nematodes and fungi with a yet elusive enzyme. Alternatively, as FGly modification has not been studied so far in these groups of organisms, it remains unknown if their sulfatases require this post-translational modification to be active.

### Sulfatases in *P. pacificus*

The sulfatase-encoding gene *eud-1* was the first gene identified to regulate mouth-form plasticity in *P. pacificus* (Ragsdale et al., 2013). In the *P. pacificus* wild type strain PS312, the majority of animals express the predatory Eu mouth-form. Therefore, genetic screens aimed at identifying *eu*rystomatous form *d*efective (*eud*) mutants that would only generate St animals. From a screen of less than 4,000 haploid genomes, 17 mutants with a *Eud* phenotype were isolated. Of these, four were dominant with heterozygous animals having a mutant all-St phenotype and all of them turned to be alleles of the same gene, *eud-1*. The cloning of *eud-1* not only revealed this gene to encode a sulfatase but also that all four mutations reduce or eliminate gene function (Ragsdale et al., 2013). These *reduction-of-function* mutations and the associated dominant phenotype indicate this gene to be haploinsufficient and to act as a developmental switch. Indeed, *eud-1* mutants can be rescued by the overexpression of a wild type copy of *eud-1*, whereas overexpression of the mutant version of *eud-1* will not cause a Eu phenotype in wild type animals (Ragsdale et al., 2013). It should be noted that such dominant *reduction-of-function* mutants are extremely rare with only one example known in *C. elegans* after more than 50 years of forward genetic screening, whereas *gain-of-function* mutants with a dominant phenotype are quite common (Hodgkin, 2005).

**FIGURE 2:**
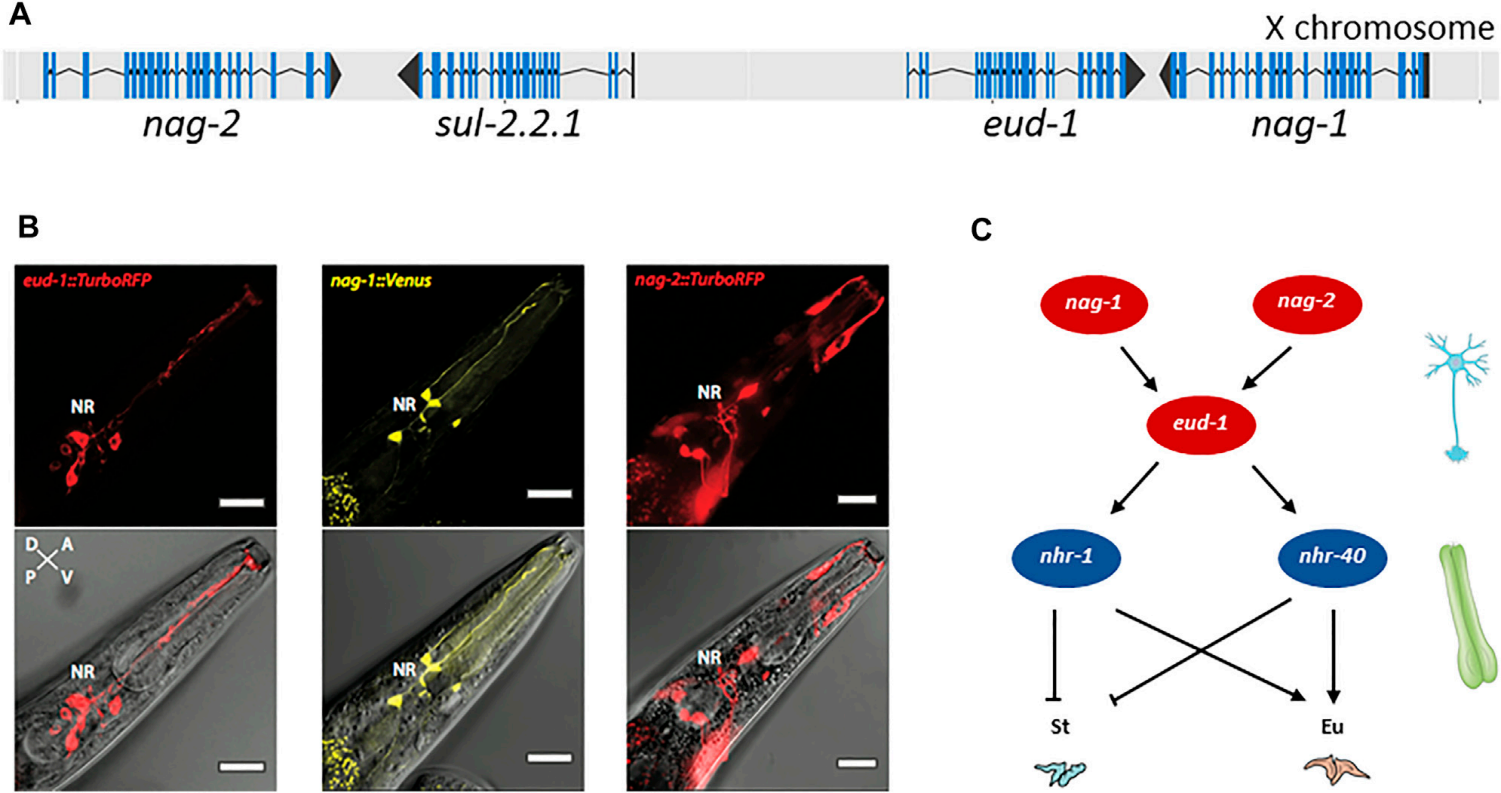
Genes involved in mouth-form dimorphism. **(A)** The switch gene *eud-1* is located on the X chromosome of *P. pacificus* in an inverted tandem configuration (head-to-head orientation) with its paralog *sul-2.2.1*. In the same gene cluster, the two sulfatase genes are surrounded by a pair of inverted and duplicated genes encoding the α-N-acetylglucosaminidases NAG-1 and NAG-2. Blue lines indicate the coding sequence (CDS); black lines represent the untranslated regions (UTRs) of the gene. Figure adapted from (Sieriebriennikov et al., 2018). **(B)** *eud-1, nag-1* and *nag-2* are expressed in distinct sensory neurons. Depicted are head sections of worms overexpressing fluorescent *eud-1, nag-1* or *nag-2* transcriptional reporters. Bottom: overlay of a DIC image with the TurboRFP (red fluorescent protein) or Venus fluorescence. Top: the same without the DIC image. Bar = 20 μm. **(C)** Partial genetic network involved in mouth-form plasticity indicating some of the key players, the α-N-acetylglucosaminidases *nag-*1 and *nag-*2, the sulfatase-encoding gene *eud-1* and the nuclear hormone receptors *nhr-40* and *nhr-1*. *nag-1*, *nag-2* and *eud-1* are expressed in sensory neurons whereas *nhr-40* and *nhr-1* expression is mainly detected in the pharyngeal muscle cells, which are thought to secrete the structural components of the teeth. St: stenostomatous; Eu: eurystomatous.

Further characterization revealed that EUD-1 might act as a sulfatase. *Ppa-*EUD-1, like *Cel-*SUL-2, is most similar to the human arylsulfatases A, GALNS and STS (Table 2). Application of sulfate and phosphate ions in the worm diet, which are known inhibitors of arylsulfatases, mimicked the *eud-1* phenotype (Ragsdale et al., 2013). As indicated above, the *P. pacificus* genome contains more sulfatase genes than the *C. elegans* genome and *eud-1* is indeed one of the genes resulting from lineage-specific gene duplications. While the arylsulfatase *Cel-sul-2* is a single copy gene on chromosome IV, there are three *sul-2*-related genes in *P. pacificus* (Table 2). The ancestral gene is also located on chromosome IV and is termed *Ppa-sul-2.1*. The two additional copies, *eud-1* and *Ppa-sul-2.2.1*, are located on the X chromosome with an interesting head to head organization and a separation by 7 kb of potential regulatory sequence (Figure 2A). *Ppa-sul-2.2.1* and *Ppa-sul-2.1* were soon shown to play no role in mouth-form specification (Ragsdale and Ivers, 2016). However, the full complexity of the *eud-1* locus was only revealed through several additional experiments. First, the conserved histone-acetyltransferase *Ppa-lsy-12* is required for *eud-1* expression suggesting that *eud-1* expression and mouth-form regulation are subject to epigenetic control (Serobyan et al., 2016). Second, an antisense-*eud-1* message exists at the locus that acts as a positive regulator of *eud-1* (Serobyan et al., 2016). Finally, two more enzyme encoding genes are part of the *eud-1* locus. These genes encode for N-acetylglucosaminidases (nag) and *nag-1* and *nag-2* are located adjacent to *sul-2.2.1* and *eud-1*, respectively (Figure 2A). Inactivation of both genes results in the opposite phenotype of *eud-1* mutants: all animals have the Eu mouth-form even in liquid culture conditions that induce the St morph in wild type animals (Sieriebriennikov et al., 2018). Interesting, *nag-1*, *nag-2* and *eud-1* are expressed in a small set of different sensory neurons suggesting that they play a key role in environmental perception (Figure 2B). Importantly, *eud-1* is epistatic over *nag-1* and *nag-2* as a quadruple knockout of the complete multigene locus has an all-St phenotype similar to *eud-1* single mutants (Sieriebriennikov et al., 2018). Thus, the sulfatase EUD-1 plays a crucial role in mouth-form regulation as the central developmental switch. First, it is involved in the sensing of the environment and second, it integrates different environmental inputs to guide final decision making of mouth-form. It is important to note that while these genetic experiments clearly show a key role of a sulfatase in regulating a plastic developmental decision, the exact biochemical function and the potential substrates remain currently unknown. It has been speculated based on the physical proximity of sulfatases and N-acetylglucosaminidases that they might act on the extracellular matrix of neurons. However, no experimental support of this hypothesis is currently available and future studies have to shed light on the actual substrates of EUD-1.

### Sulfotransferases in *P. pacificus*

Further genetic studies have identified a complex gene regulatory network (GRN) to control mouth-form plasticity with *eud-1* representing the key developmental switch (Figure 2C) (Sieriebriennikov et al., 2020). The identification of this GRN was in large parts based on the strong all-St phenotype of the *eud-1* mutant itself, which allowed unbiased genetic screens for suppressors resulting in all-Eu animals.

First, three suppressor alleles with an all-Eu phenotype were shown to result from mutations in the nuclear hormone receptor *Ppa-nhr-40* (Kieninger et al., 2016) and were later on shown to result from *gain-of-function* mutations (Sieriebriennikov et al., 2020). In contrast, the *loss-of-function* phenotype of *Ppa-nhr-40* is all-St and thus, similar to the *eud-1* mutant phenotype (Sieriebriennikov et al., 2020). Also, *loss-of-function* mutations in another nuclear hormone receptor, *Ppa-nhr-1*, have an all-St phenotype, but with additional morphological defects in teeth formation unknown from other mutants (Sieriebriennikov et al., 2020). These studies would be compatible with the EUD-1 protein regulating the sulfation state of potential ligands of nuclear hormone receptors. Note that while nematodes have undergone a substantial expansion of nuclear hormone receptors with more than 250 genes in *P. pacificus* and *C. elegans*, only one ligand has been identified: the steroid hormone dafachronic acid that regulates the nuclear hormone receptor DAF-12 in multiple nematode species (Motola et al., 2006; Ogawa et al., 2009). Thus, the ligands of nematode nuclear hormone receptors remain little understood and similarly, nothing is known about a potential role of sulfation in the regulation of such ligands.

Second, the same genetic screen for *eud-1* suppressors resulted in the identification of a sulfotransferase, mutations in which also produced an all-Eu phenotype and thus were named *seud-1* (*s*uppressor of *eud-1*) (Bui et al., 2018). Transgenic overexpression of *seud-1* revealed the sulfotransferase to be dosage-dependent, similar to *eud-1* (Bui et al., 2018). When mutants of *eud-1* and *seud-1* were used to produce all types of homozygous and heterozygous combinations, a graduation of phenotypes was observed. For example, animals with one copy of the sulfotransferase *seud-1* and two copies of the sulfatase *eud-1* were Eu (Bui et al., 2018). Besides the work of Bui and co-workers, an independent study resulted in the identification of the same sulfotransferase. A pharmacological screen found bisphenol A to induce the St mouth phenotype in wild type animals (Namdeo et al., 2018). As already indicated above, in *C. elegans* the sulfotransferase *ssu-1* sulfates bisphenol A (Hattori et al., 2006). Indeed, a systematic screen of the five sulfotransferases of *P. pacificus* that are most similar in sequence to *Cel-ssu-1*, identified a single gene, named *sult-1*, to control mouth-form plasticity (Namdeo et al., 2018). Note that these independent studies have named the same sulfotransferase gene as *seud-1* and *sult-1*. Importantly, the expression of SEUD-1/SULT-1 (pharyngeal muscle cells) is distinct from EUD-1 (sensory neurons) and therefore, it is possible that two independent sulfation processes regulate mouth-form plasticity (Bui et al., 2018; Namdeo et al., 2018).

### Sulfolipids of P*. pacificus* Induce Escape Behavior in *C. elegans*

Completely independent insight pointing towards the role of sulfated molecules in the biology of *P. pacificus* nematodes came from studies in analytical chemistry. The predatory behavior of *P. pacificus* is thought to be of importance in the context of the ecology of this nematode. While *P. pacificus* is a soil nematode, it is most reliably found in association with adult scarab beetles, *i.e.* cock chafers, stag beetles and dung beetles. On the living beetle, nematodes are found in the dauer stage, the arrested and stress-resistant, alternative larval stage that can survive long periods of harmful conditions. It is important to note that several, unrelated nematode species can be found on beetles and also other insects, and many of them wait for their vector to die. Indeed, after the beetles’ death back in the soil, bacteria and fungi will grow on the carcass. These food signals will cause the exit of the nematodes from the dauer stage and they rapidly mature to feed and reproduce on this time-limited food source. Indeed, recent studies in *P. pacificus* have indicated the dynamics and succession of nematodes on beetle carcasses in the context of the microbiome of the insect cadaver (Meyer et al., 2017; Renahan et al., 2021; Renahan and Sommer, 2021). The predatory behavior of Eu animals of *P. pacificus* is thought to serve a dual role: on the one hand, it allows supplementation of its bacterial diet by preying on nematodes; on the other hand, it will result in the elimination of competitors for the limited bacterial food available. This phenomenon is known as intraguild predation and represents an important concept in ecology (Polis and Holt, 1992; Quach and Chalasani, 2020).

While there is no obvious evidence that *P. pacificus* and *C. elegans* share the same ecosystem, a study by Liu and co-workers indicated a defensive response of *C. elegans* when it is exposed to *P. pacificus* predators (Liu et al., 2018). Subsequent studies revealed the behavioral details of this predator-prey competition (Quach and Chalasani, 2020). Strikingly, the original defensive response of *C. elegans* larvae towards *P. pacificus* is caused by predator-secreted sulfolipids. Specifically, fractionation of the *P. pacificus* secretome and analytical chemistry identified several (w-1)-branched-chain sulfolipids to induce the escape behavior in *C. elegans* prey (Liu et al., 2018). These findings are consistent with previous studies that showed an avoidance behavior of *C. elegans* against sodium dodecyl sulfate (SDS) (Hilliard et al., 2004). Importantly, the production and secretion of branched chain fatty acids is specific to *P. pacificus* and likely its relatives (Liu et al., 2018). The presence of multiple secreted sulfolipids in *P. pacificus* might open the door to several independent functions in the organism. For example, it might well be that branched chain fatty acids can serve as ligands for nuclear hormone receptors although no functional studies are yet available to support this hypothesis. In conclusion, genetic and chemical studies provide strong support for the role of sulfation in the development and the behavior of this nematode.

### Open Questions and Future Perspectives

The literature described in this review highlights the significance of sulfation and desulfation pathways during development of different nematodes. From neuronal organization, to the regulation of phenotypic traits and behavior, sulfatases and sulfotransferases appear to modify a wide range of substrates also in nematodes. Despite the work done so far, we still possess a limited understanding on the localization, substrates and regulation of the panoply of sulfation-associated enzymes present in worms. Many of the sulfotransferases and sulfatases remain uncharacterized; their substrates are unknown, we have no understanding of their biological significance or subcellular localization, and we lack defined knowledge on their pattern of expression. Moreover, specific sulfated molecules, such as sulfolipids, act as signaling molecules between animals (Quach and Chalasani, 2020). However, we still do not understand the molecular mechanisms that generate, interpret, and execute, or the universality of this communication pathway.

An exciting research area in sulfation relates to the regulation of the enzymatic activity by post-translational modifications, such as FGly and glycosylation. As mentioned above, nematodes lack a recognizable FGE. Future studies that aim at detecting this unique modification in the proteome of nematodes, the corresponding modifying enzyme and its mechanism of action have great potential to elucidate alternative systems that introduce the FGly modification in eukaryotic sulfatases.

The significance of sulfation pathways in parasitic nematodes, like *S. ratti*, has never been addressed. With the development of specific sulfatase inhibitors, it is therefore of great importance to study their impact on the infection and ability of the parasite to invade the host. A deeper understanding on the sulfation players and their functions in these pathogenic worms may lead to improved therapeutic strategies that aim at eliminating the pathogen from their natural host. We are therefore looking forward to use nematodes in the quest to obtain a comprehensive understanding of the mechanisms and roles that sulfation plays in organismal development.
